# Establishing thresholds and parameters for pandemic influenza severity assessment, Australia

**DOI:** 10.2471/BLT.18.211508

**Published:** 2018-06-25

**Authors:** Kaitlyn Vette, Christina Bareja, Robert Clark, Aparna Lal

**Affiliations:** a The National Centre for Epidemiology and Population Health, Australian National University, Canberra, ACT 2600, Australia.; b Office of Health Protection, Australian Government Department of Health, Canberra, Australia.; c Statistical Consulting Unit, Australian National University, Canberra, Australia.

## Abstract

**Objective:**

To implement the World Health Organization’s pandemic influenza severity assessment tool in Australia, using multiple sources of data to establish thresholds and measure influenza severity indicators.

**Methods:**

We used data from four reliable sources: sentinel general practitioner surveillance, hospital surveillance, a public health hotline and an influenza-like illness survey system. We measured three influenza severity indicators (transmissibility, impact and disease seriousness) defined using pandemic influenza severity assessment guidelines. We used the moving epidemic method and a seriousness indicator-specific method to set thresholds for indicator parameters using 2012–2016 data. We then applied the thresholds to data from the 2017 influenza season.

**Findings:**

We were able to measure and produce thresholds for each severity indicator. At least one laboratory-confirmed influenza parameter was used to measure each indicator. When thresholds were applied to the 2017 season, there was good agreement across all data sources in measuring activity for each indicator. The season was characterized as having high transmissibility and extraordinary impact. Seriousness was characterized as moderate overall and in all age groups except those aged ≥ 65 years for whom it was high. This matched the description of the season produced by the Australian national influenza surveillance committee, based on expert opinion and historical ranges.

**Conclusion:**

The pandemic influenza severity assessment and moving epidemic method provide a robust and flexible method to enable an evidence-based assessment of seasonal influenza severity across diverse data sources. This is useful for national assessment and will contribute to global monitoring and response to circulating influenza with pandemic potential.

## Résumé

**Objectif:**

Mettre en œuvre l'outil d'évaluation de la sévérité de la grippe pandémique de l'Organisation mondiale de la Santé en Australie, à l'aide de plusieurs sources de données, pour établir des seuils et mesurer des indicateurs de la sévérité de la grippe.

**Méthodes:**

Nous avons utilisé des données tirées de quatre sources fiables: surveillance sentinelle des médecins généralistes, surveillance hospitalière, permanence téléphonique en matière de santé publique et système d'enquête sur le syndrome de type grippal. Nous avons mesuré trois indicateurs de la sévérité de la grippe (transmissibilité, impact et sévérité de la maladie) définis à l'aide du guide pour évaluer la sévérité de la grippe pandémique. Nous avons utilisé la Moving Epidemic Method et une méthode propre à chaque indicateur de sévérité afin de définir des seuils pour les paramètres des indicateurs à l'aide de données de 2012-2016. Nous avons ensuite appliqué ces seuils aux données de la saison 2017 de la grippe.

**Résultats:**

Nous avons pu mesurer et établir des seuils pour chaque indicateur de la sévérité. Au moins un paramètre de la grippe confirmé en laboratoire a été utilisé pour mesurer chaque indicateur. Lorsque les seuils ont été appliqués à la saison 2017, une forte concordance a été constatée entre toutes les sources de données concernant la mesure de l'activité pour chaque indicateur. La saison a été caractérisée par une transmissibilité élevée et un impact extraordinaire. La sévérité a été définie comme étant modérée dans l'ensemble et dans tous les groupes d'âge, à l'exception du groupe des ≥ 65 ans pour lequel elle a été définie comme étant élevée. Cela correspondait à la description de la saison faite par le comité national australien de surveillance de la grippe, à partir de l'opinion d'experts et de plages historiques.

**Conclusion:**

L'évaluation de la sévérité de la grippe pandémique et la Moving Epidemic Method fournissent une méthode fiable et souple pour permettre une évaluation fondée sur des données scientifiques de la sévérité de la grippe saisonnière à partir de diverses sources de données. Cela est utile pour l'évaluation nationale et contribuera au suivi mondial et à la lutte contre les virus grippaux circulants à potentiel pandémique.****

## Resumen

**Objetivo:**

Implementar la herramienta de evaluación de la gravedad de la gripe pandémica de la Organización Mundial de la Salud en Australia, utilizando múltiples fuentes de datos para establecer umbrales y medir los indicadores de gravedad de la gripe.

**Métodos:**

Se utilizaron datos de cuatro fuentes fiables: la vigilancia de médicos generales centinelas, la vigilancia hospitalaria, una línea telefónica de salud pública y un sistema de encuestas sobre enfermedades similares a la gripe. Se midieron tres indicadores de gravedad de la gripe (transmisibilidad, impacto y gravedad de la enfermedad) definidos mediante las pautas de la Evaluación de la gravedad de la gripe pandémica. Se siguió el método de epidemia móvil y un método específico de indicador de gravedad para establecer umbrales para los parámetros de los indicadores con los datos de 2012-2016. A continuación, se aplicaron los umbrales a los datos de la temporada de gripe 2017.

**Resultados:**

Se midieron y crearon umbrales para cada indicador de gravedad. Se utilizó al menos un parámetro de gripe confirmado por laboratorio para medir cada indicador. Cuando se aplicaron los umbrales a la temporada 2017, todas las fuentes de datos coincidieron en medir la actividad de cada indicador. La temporada se caracterizó por una alta transmisibilidad y un impacto extraordinario. La gravedad fue moderada en general y en todos los grupos de edad, excepto para los de ≥ 65 años, para los que fue alta. Esto coincidió con la descripción de la temporada elaborada por el comité nacional australiano de vigilancia de la gripe, basada en la opinión de los expertos y en los rangos históricos.

**Conclusión:**

La Evaluación de la gravedad de la gripe pandémica y el método de epidemia móvil proporcionan un método robusto y flexible para permitir una evaluación basada en la evidencia de la gravedad de la gripe estacional a través de diversas fuentes de datos. Esto es útil para la evaluación nacional y contribuirá a la vigilancia y respuesta mundial a la gripe circulante con potencial pandémico.

## ملخص

**الغرض:**

تنفيذ أداة تقييم شدة الإنفلونزا الوبائية، التابعة لمنظمة الصحة العالمية في أستراليا، باستخدام مصادر متعددة للبيانات بهدف تحديد العتبات وقياس مؤشرات شدة الإنفلونزا.

**الطريقة:**

استخدمنا البيانات من أربعة مصادر موثوق بها: مراقبة الممارس العام، ومراقبة المستشفى، والخط الساخن للصحة العامة، ونظام مسح الأمراض المشابهة للإنفلونزا. قمنا بقياس ثلاثة مؤشرات لشدة الإنفلونزا (القابلية للانتشار، والتأثير، وخطورة المرض)، والتي تم تحديدها باستخدام المبادئ التوجيهية لتقييم شدة الإنفلونزا الوبائية. استخدمنا طريقة انتشار الوباء، وطريقة مؤشر الخطورة، لتحديد عتبات معلمات المؤشر باستخدام بيانات 2012 إلى 2016. ثم قمنا بتطبيق العتبات على البيانات من موسم الإنفلونزا لعام 2017.

**النتائج:**

كان بمقدورنا قياس وإنتاج عتبات لكل مؤشر شدة. تم استخدام معامل واحد على الأقل للإنفلونزا معزز من جانب المختبر، لقياس كل مؤشر. عندما تم تطبيق العتبات على موسم 2017، كان هناك اتفاق جيد بين جميع مصادر البيانات على قياس النشاط لكل مؤشر. وقد تم وصف هذا الموسم بأنه ذو قابلية عالية للتفشي، وله تأثير غير عادي. وتم وصف الخطورة بأنها معتدلة بشكل عام، وفي جميع الفئات العمرية، باستثناء أولئك الذين تبلغ أعمارهم 65 سنة أو تزيد، حيث كانت الخطورة لديهم مرتفعة. وقد تطابق هذا مع وصف الموسم الذي أصدرته لجنة مراقبة الإنفلونزا الوطنية الأسترالية، بناءً على آراء الخبراء والنطاقات التاريخية.

**الاستنتاج:**

يوفر كل من تقييم خطورة الأنفلونزا الوبائية، وطريقة انتشار الوباء، طريقة فعالة ومرنة للتمكين من إجراء تقييم قائم على الأدلة لشدة الإنفلونزا الموسمية عبر مصادر متنوعة للبيانات. ويعد هذا مفيداً للتقييم الوطني، وسوف يسهم في الرصد العالمي، والاستجابة للإنفلونزا المتفشية المحتمل تحولها لوباء. ****

## 摘要

**目的:**

旨在在澳大利亚实施世界卫生组织的大流行性流感严重程度评估工具，使用多种来源的数据以确定阈值并衡量流感严重程度指标。

**方法:**

我们使用来自四个可靠来源的数据：定点全科医生监测、医院监测、公共卫生热线和流感样疾病调查系统。我们衡量了使用大流行性流感严重程度评估指南定义的三种流感严重程度指标（传染性、影响和疾病严重程度）。我们使用 2012 至 2016 年数据，采用流行疫情方法和严重程度指标特定方法来设置指标参数的阈值。然后，将这些阈值应用于 2017 年流感季节的数据。

**结果:**

我们衡量并且生成每个严重程度指标的阈值。至少有一个实验室确认的流感参数被用来衡量各指标。当阈值应用于 2017 年季节时，所有数据来源在各指标的衡量活动中都达成了很好的一致性。该季节的特点是具有高度传染性和特别的影响。除了那些年龄≥65 岁的人群以外，各年龄段人群的严重程度均为中等程度。根据专家意见和历史范围，这与澳大利亚国家流感监测委员会提供的季度说明相匹配。

**结论:**

大流行性流感严重程度评估和流行疫情方法提供了一种稳健而灵活的方法，可以对不同数据来源的季节性流感严重程度进行循证评估。这对全国性评估是有用的，并将有助于全球监测和应对可能引起大流行的流行性感冒。****

## Резюме

**Цель:**

Внедрить в Австралии инструмент Всемирной организации здравоохранения для оценки степени тяжести пандемического гриппа с использованием нескольких источников данных для определения пороговых значений и показателей степени тяжести гриппа.

**Методы:**

Авторы использовали данные из четырех надежных источников: дозорного эпиднадзора на уровне врачей общей практики и больниц, горячей линии общественного здравоохранения и системы эпиднадзора за гриппоподобными заболеваниями. Были определены три показателя степени тяжести гриппа (контагиозность, воздействие и серьезность заболевания), определяемые с помощью руководства для оценки степени тяжести пандемического гриппа. Авторы применили метод, основанный на движении эпидемии, а также метод, основанный на показателе серьезности заболевания, чтобы определить пороговые значения для параметров с использованием данных за 2012–2016 гг. Затем полученные пороговые значения были применены к данным по гриппу сезона 2017 года.

**Результаты:**

Авторы смогли измерить и создать пороговые значения для каждого показателя степени тяжести. Для измерения каждого показателя использовался по крайней мере один лабораторно подтвержденный параметр гриппа. После применения пороговых значений к сезону 2017 года при измерении активности по каждому показателю наблюдалась хорошая согласованность данных среди всех источников. Сезон был охарактеризован как имеющий высокую контагиозность и чрезвычайно сильное воздействие. Серьезность характеризовалась как умеренная в целом и во всех возрастных группах, за исключением лиц в возрасте ≥ 65 лет, для которых она была высокой. Это соответствовало описанию сезона, подготовленного Австралийским национальным комитетом по эпиднадзору за гриппом, которое основано на экспертном заключении и исторических диапазонах.

**Вывод:**

Оценка степени тяжести пандемического гриппа и метод, основанный на движении эпидемии, обеспечивают надежный и гибкий способ, позволяющий провести достоверную оценку степени тяжести сезонного гриппа, используя различные источники данных. Это будет способствовать оценке на национальном уровне, а также глобальному мониторингу и реагированию на распространение гриппа с пандемическим потенциалом.****

## Introduction

Influenza is an important global health challenge, with an estimated 10–20% of the 7.6 billion world population infected annually.^1^ Due to the changing presentation of epidemics, the beginning, duration and severity of influenza seasons are difficult to predict.^2^ Accurately understanding these factors can inform the timing, focus and scale of public health action.^3^ In Australia, national influenza surveillance currently uses historical ranges and expert opinion to determine the start of the season, its severity and whether disease activity is within the expected range.^4^

In 2011, the World Health Organization (WHO) reviewed the response to the 2009 influenza pandemic.^5^ The report identified a major challenge in measuring global influenza severity due to differences in population health and services, a lack of comparable data and unstandardized measurements. As a result, the WHO developed the pandemic influenza severity assessment, a tool to enhance influenza surveillance and standardize severity reporting by Member States, with the goal of enabling a global, real-time assessment of influenza severity.^3^ The tool defines the severity of influenza using three indicators: (i) transmissibility, (ii) seriousness of disease and (iii) impact.^3^ The tool suggests data parameters to measure each severity indicator and recommends a variety of methods to determine thresholds for the parameters. 

The pandemic influenza severity assessment recommends that transmissibility and impact indicators are measured using weekly rates, and that disease seriousness is measured using a cumulative value. As such, thresholds for the different measurements are calculated using separate methods. We chose the moving epidemic to calculate transmissibility and impact thresholds,^6^^,^^7^ and a seriousness indicator-specific method to set thresholds for seriousness of disease.^3^ The moving epidemic method is a tool for calculation of influenza activity thresholds based the epidemic curves of historical data. The method has been piloted by WHO and the European Centre for Disease Prevention and Control and has been applied in countries including Spain, the United Kingdom of Great Britain and Northern Ireland and the United States of America.^7^^–^^10^ In Australia, thresholds for influenza surveillance have previously been established in one jurisdiction, but have not been used in national influenza assessments.^11^^,^^12^

In this study we applied the pandemic influenza severity assessment to select appropriate surveillance parameters and set thresholds for assessment of seasonal influenza severity in Australia. In doing so, we sought to demonstrate a country-level implementation of the tool and to create an evidence-based, standardized assessment of influenza severity across Australia that would contribute to the WHO’s global severity assessment.

## Methods

### Data sources

We determined a range of data sources capable of measuring the three influenza severity indicators: transmissibility, disease seriousness and impact. For each indicator we chose at least two sources of Australian surveillance data to calculate the relevant parameters, including one source of laboratory-confirmed influenza data (Table 1). Four data sources were used in total. Flutracking is a survey-based system that conducts weekly surveillance of influenza-like illness (ILI) in the community from approximately 27 000 participants in all Australian jurisdictions.^4^ The public health hotline Healthdirect collects surveillance data on ILI from callers in all jurisdictions except the states of Victoria and Queensland, receiving approximately 640 000 calls annually. The Australian sentinel practices research network is a sentinel general practitioner surveillance system that collects data on consultations for ILI and systematically swab-tests these patients for influenza virus at approximately 200 sites across all jurisdictions. The influenza complications alert network is a sentinel hospital surveillance system that collects information on confirmed cases of influenza and intensive care unit admissions from 17 hospitals in all jurisdictions.

**Table 1:** Australian data sources and parameters used to measure influenza severity indicators

Severity indicator,^a^ by data source	Type of surveillance	Parameter
**Transmissibility**		
Flutracking	Survey-based weekly surveillance of ILI in the community	Number of people reporting ILI per 1000 survey participants
Healthdirect	Callers to public health hotline	Number of callers reporting ILI per 1000 callers to hotline
Australian sentinel practices research network	Sentinel general practitioner surveillance system	(Number of people with ILI per 1000 general practitioner consultations) x (% of systematic ILI swabs confirmed positive for influenza virus)
**Impact**		
Flutracking	Survey-based weekly surveillance of ILI in the community	Number of people absent from regular duties per 1000 survey participants with ILI
Influenza complications alert network	Sentinel hospital surveillance system	Number of laboratory-confirmed influenza admissions per 1000 available hospital beds
**Seriousness of disease**		
Influenza complications alert network	Sentinel hospital surveillance system	Cumulative number of intensive care unit admissions per 100 laboratory-confirmed influenza admissions
Healthdirect	Callers to public health hotline	Cumulative number of callers with ILI advised to seek urgent medical attention per 1000 callers with ILI (split into age groups: < 15, 15–64 and ≥ 65 years)

ILI: influenza-like illness.

^a^ Influenza severity indicators are from the World Health Organization pandemic influenza severity assessment tool.^3^ Transmissibility measures how many people in a population get sick from influenza on a weekly basis. Seriousness of disease measures how severely sick individual people get when infected with the influenza virus. Impact measures how the influenza epidemic or pandemic affects society, including the health-care system.

We obtained weekly incidence data for the years 2012–2017 from each surveillance system; there were no missing data. Routine case definitions specific to each system were used (Table 1).

### Data analysis

We used data from 2012–2016 to determine thresholds for week numbers 18–39 and then applied them to the 2017 season. We assigned week numbers using the International Organization for Standardization 8601 standard.

#### Moving epidemic method

To determine the thresholds for the transmissibility and impact parameters we used the moving epidemic method, which models historical weekly rates of influenza activity to determine when the epidemic period is likely to occur and to quantify expected activity levels.^6^ We calculated pre-and post-epidemic thresholds, as well as thresholds for moderate, high and extraordinary influenza intensity for each parameter. For threshold calculations we used R software, version 3.4.1 (R Foundation for Statistical Computing, Vienna, Austria)^13^ and the moving epidemic method R package, version 2.9 (Health Sentinel Network of Castilla y León, Valladolid, Spain).^14^

To create thresholds, the moving epidemic method function *memmodel* was used. Within *memmodel*, there are multiple function options to select from, including the confidence interval levels from which to calculate thresholds; how many seasons of data to use; and how to determine the timing of the epidemic period based on temporal trends (referred to as the optimal timing). We chose the slope method to determine the optimal timing of the epidemic period, which calculates the curve of the minimum number of consecutive weeks with the maximum accumulated proportion of the parameter rates and matches this to the mean slope.^14^ The moving epidemic method package recommends the fixed criterium method (matching the maximum accumulated proportion of rates to a predetermined parameter value) to determine the epidemic period.^14^ However, the slopes of the epidemic curves in our data were not steep enough to use this method with the predefined parameter value set by the package, based on European data. We generated a data-specific parameter value for one data source, and when we used it in the fixed criterium method we found that this produced near-identical results as using the predefined parameter value and the slope method. We therefore used the slope method for subsequent analyses. Within *memmodel*, we calculated the pre- and post-epidemic thresholds using the one-sided 95% point confidence interval (CI) of the arithmetic mean. We set pre-epidemic values for threshold calculation at −1, allowing the appropriate number of points determined by the number of influenza seasons available. We calculated intensity thresholds using the geometric mean and one-sided point CIs. We used upper limits of the 40%, 90% and 99% CIs for moderate-, high- and extraordinary-intensity thresholds. For other calculations within *memmodel* we determined the median and its two-sided CI using the KC method (an adaptation of K.C. Carrière’s CI calculation using quantiles).^14^^,^^15^

#### Seriousness method 

As recommended in the pandemic influenza severity assessment, we used a WHO method specific to influenza seriousness to calculate thresholds for this indicator. To determine the thresholds for moderate, high and extraordinary disease seriousness we used the mean, mean plus 1 standard deviation and mean plus 3 standard deviations of end-of-season values. We analysed data for the seriousness parameters in three age groups (< 15, 15–64 and ≥ 65 years). Calculating an epidemic threshold for this parameter is not recommended, as the measurement is cumulative and stabilizes after the peak of the season. We calculated the seriousness parameter thresholds using Office Excel 2010 (Microsoft Corp., Redmond, United States of America).

## Results

We calculated the weekly mean of the 2012–2016 data and weekly 2017 values for each parameter from week 18–39 or until the post-epidemic threshold was crossed. Detailed interpretations of each data source have been published elsewhere.^4^^,^^16^

### Transmissibility

**Fig. 1:**
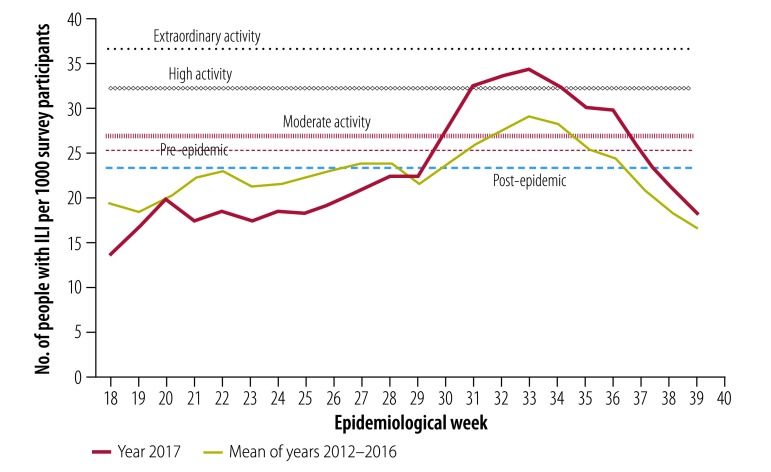
Influenza transmissibility activity by epidemiological week, Australia, 2017

Using data from the Flutracking community surveillance system, the thresholds for moderate, high and extraordinary disease transmission were set at 26.8, 32.2 and 36.5 people with ILI per 1000 survey participants, respectively (Fig. 1). The pre- and post-epidemic thresholds were set at 25.4 and 23.4 per 1000, respectively. Transmission in the 2017 season peaked at 34.3 per 1000 in week 33, reaching the definition of high influenza activity.

ILI: influenza-like illness.

Notes: Thresholds for moderate, high and extraordinary levels of influenza activity were the 40%, 90% and 99% confidence intervals of the geometric mean, respectively. We used data from Flutracking, which conducts weekly surveillance of ILI in the community from approximately 27 000 participants.

**Fig. 2:**
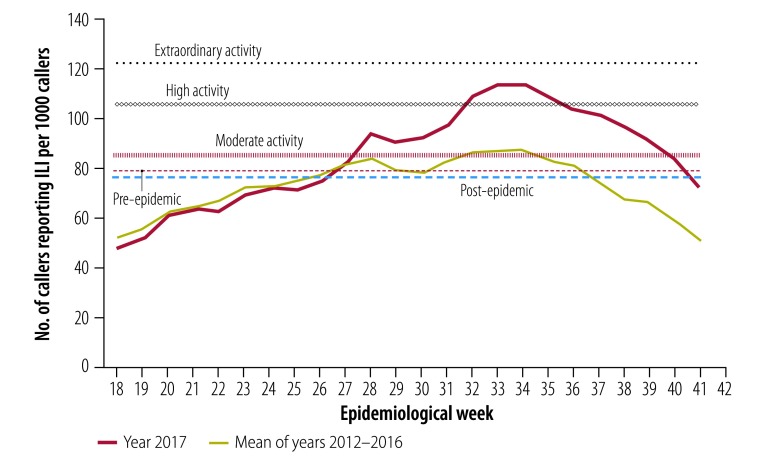
Influenza transmissibility activity by epidemiological week, Australia, 2017

Data from the Healthdirect public health hotline produced thresholds for moderate, high and extraordinary transmission of 85.8, 105.8 and 122.0 people with ILI per 1000 callers to the hotline, respectively (Fig. 2). The pre-epidemic and post-epidemic thresholds were set at 78.8 and 76.6 per 1000, respectively. The 2017 season peaked at 113.5 per 1000 (high activity) in week 34.

ILI: influenza-like illness.

Notes: Thresholds for moderate, high and extraordinary levels of influenza activity were the 40%, 90% and 99% confidence intervals of the geometric mean, respectively. We used data from Healthdirect public health hotline, which collects surveillance data on ILI, receiving approximately 640 000 calls annually.

**Fig. 3:**
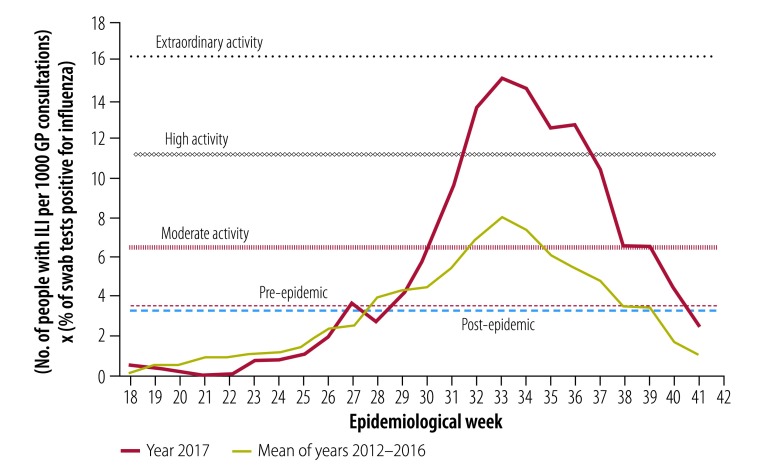
Influenza transmissibility activity by epidemiological week, Australia, 2017

GP: general practitioner; ILI: influenza-like illness.

Notes: Thresholds for moderate, high and extraordinary levels of influenza activity were the 40%, 90% and 99% confidence intervals of the geometric mean, respectively. We used data from ASPREN (Australian sentinel practices research network), which is a sentinel general practitioner surveillance system that collects data on consultations for ILI, including systematic testing for influenza virus in swabs from ILI patients consulting, at approximately 200 sites. The data are a composite measure: (no. of people with ILI per 1000 general practitioner consultations) x (% of systematic ILI swabs confirmed positive for influenza virus).

Surveillance data from the Australian sentinel practices research network provided a composite measure of transmissibility: (number of people with ILI per 1000 general practitioner consultations) × (number of swabs testing positive for influenza per 100 swabs of people with influenza like illness tested). The thresholds for moderate, high and extraordinary transmission were calculated to be 6.5, 11.2 and 16.2, respectively (Fig. 3). The pre-epidemic and post-epidemic thresholds were set at 3.4 and 3.5, respectively. The peak of the 2017 season was 15.1 (high activity) in week 33.

### Impact

**Fig. 4:**
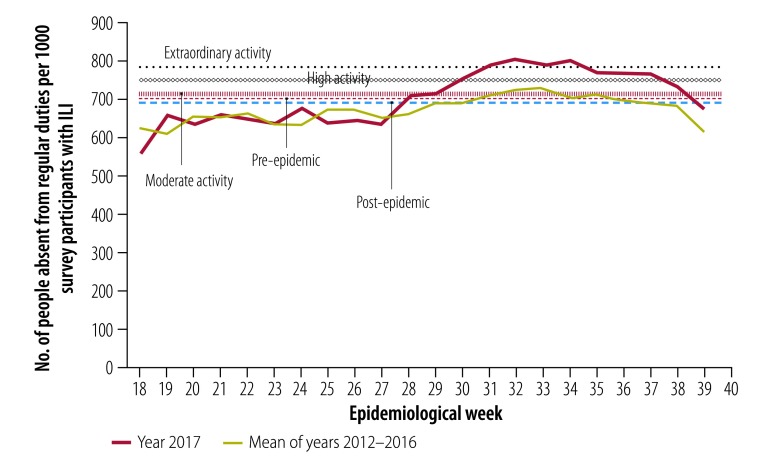
Influenza impact activity by epidemiological week, Australia, 2017

The thresholds for moderate, high and extraordinary disease impact from the Flutracking system were 713.5, 753.2 and 781.5 people with ILI absent from normal duties per 1000 survey participants with ILI, respectively (Fig. 4). The 2017 season peaked at 796.8 per 1000 in week 32, defined as extraordinary influenza impact.

ILI: influenza-like illness.

Notes: Thresholds for moderate, high and extraordinary levels of influenza activity were the 40%, 90% and 99% confidence intervals of the geometric mean, respectively. We used data from Flutracking, which is a survey-based system that conducts weekly surveillance of ILI in the community from approximately 27 000 participants.

**Fig. 5:**
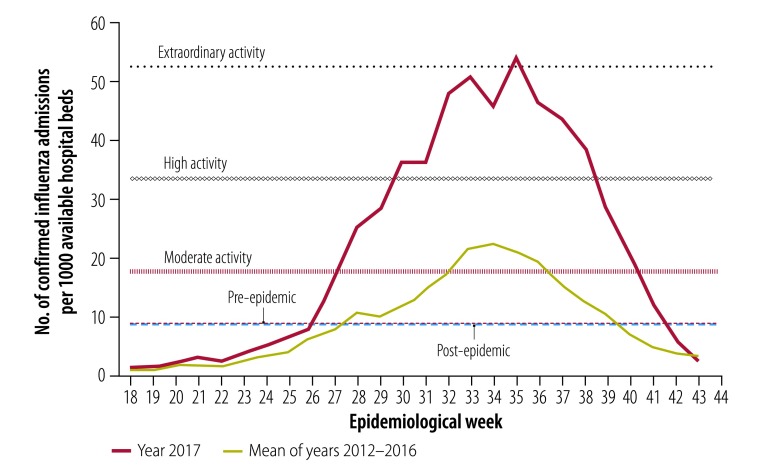
Influenza impact activity by epidemiological week, Australia, 2017

Notes: Thresholds for moderate, high and extraordinary levels of influenza activity were the 40%, 90% and 99% confidence intervals of the geometric mean, respectively. We used data from FluCAN (influenza complications alert network), which is a sentinel hospital surveillance system that collects information on confirmed cases of influenza from 17 hospitals.

Data from the influenza complications alert network, a hospital surveillance system, produced thresholds for moderate, high and extraordinary impact of 17.5, 33.6 and 52.5 hospital admissions for influenza per 1000 hospital beds, respectively (Fig. 5). The peak of the 2017 activity was 53.5 per 1000 (extraordinary impact) in week 35.

### Seriousness of disease

**Fig. 6:**
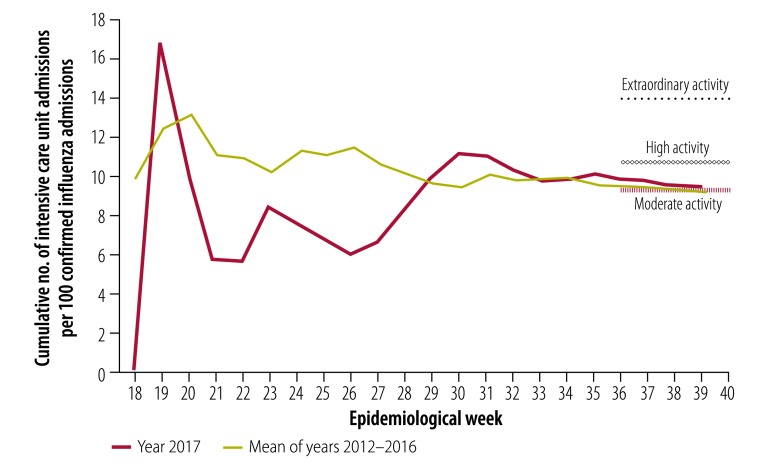
Influenza disease seriousness activity by epidemiological week, Australia, 2017

Notes: Thresholds for moderate, high and extraordinary levels of influenza activity were the mean, mean plus 1 standard deviation and mean plus 3 standard deviations of end-of-season values, respectively. Threshold lines start at 36 weeks as seriousness was not assessed until after the peak of the influenza season. We used data from FluCAN (influenza complications alert network), which is a sentinel hospital surveillance system that collects information on confirmed cases of influenza from 17 hospitals.

When the influenza complications alert network data were separated into age groups for the measurement of disease seriousness, the cumulative numerator was less than 10 at the peak of the season for some years; this made the standard error large and impractical to use for measuring seriousness. When analysed without age groupings, the thresholds for moderate, high and extraordinary seriousness were set at 9.2, 10.7 and 13.9 intensive care unit admissions per 100 cumulative hospital admissions for influenza, respectively (Fig. 6). At the end of the 2017 season, intensive care admissions were 9.4 per 100, defined as moderate influenza seriousness.

When analysed overall, thresholds for moderate, high and extraordinary seriousness from the Healthdirect system were set at 120.9, 143.0 and 187.0 callers advised to seek urgent medical attention per 1000 cumulative callers with ILI, respectively. The 2017 season ended at 124.2 urgent calls per 1000 (moderate seriousness).

**Fig. 7:**
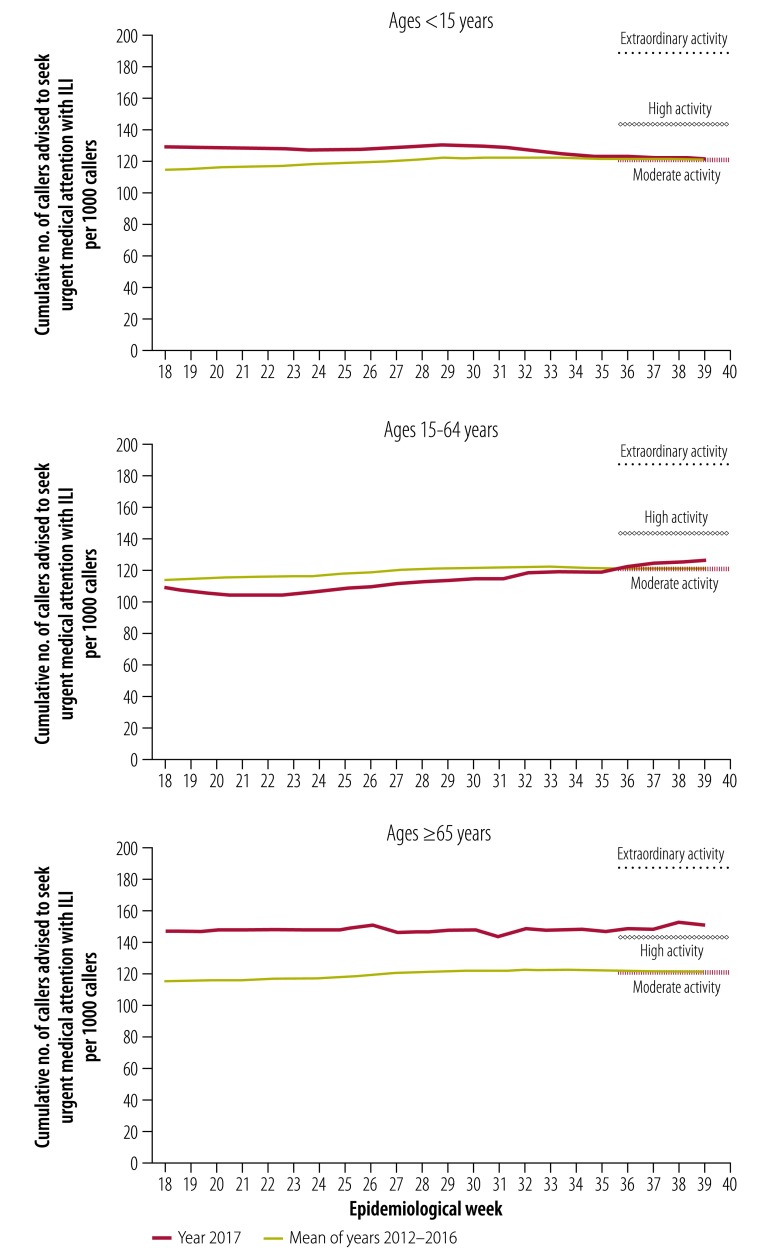
Influenza seriousness activity by epidemiological week for three age groups, Australia, 2017

In the youngest age group, Healthdirect thresholds for moderate, high and extraordinary seriousness were set at 121.0, 143.2 and 187.5 urgent callers per 1000 cumulative callers aged < 15 years with ILI, respectively (Fig. 7). At the end of the 2017 season there were 121.3 urgent calls per 1000 callers (moderate seriousness). For the middle age group, thresholds for moderate, high and extraordinary seriousness were set at 120.9, 142.9 and 187.0 per 1000 cumulative callers aged 15–64 years, respectively (Fig. 7). The 2017 season ended with 126.3 urgent calls per 1000 (moderate seriousness). For the oldest age group, thresholds for moderate, high and extraordinary seriousness were set at 120.9, 143.0 and 187.0 urgent calls per 1000 cumulative callers aged ≥ 65 years, respectively (Fig. 7). At the end of the 2017 season there were 151 urgent calls per 1000 (high seriousness).

ILI: influenza-like illness.

Notes: Thresholds for moderate, high and extraordinary levels of influenza activity were the mean, mean plus 1 standard deviation and mean plus 3 standard deviations of end-of-season values, respectively. Threshold lines start at 36 weeks as seriousness was not assessed until after the peak of the influenza season. We used data from Healthdirect public health hotline, which collects surveillance data on ILI, receiving approximately 640 000 calls annually.

## Discussion

This study has established a standardized assessment of national influenza severity using parameters recommended by the pandemic influenza severity assessment.^3^ We were able to measure each severity indicator with two independent sources of data, one of which used laboratory-confirmed influenza data to validate ILI measurements. Standardized disease intensity and epidemic thresholds using existing data sources provide a practical, country-specific, yet internationally reportable, method of assessing influenza severity.

The data sources used in this study have been formally evaluated to have moderate to high timeliness, reliability and data quality (Wright R, Australian National University, unpublished data, 2017; Schicker RF, United States Centers for Disease Control and Prevention, unpublished data, 2016).^17^^,^^18^ While each source inherently captures data from different geographical areas and demographic groups, they are used as complementary components in routine national surveillance in Australia.^4^^,^^16^ Fortnightly seasonal analysis has found that the systems detect common trends in influenza activity.^19^ Our study also demonstrated conformity across data sources in the measurement of influenza trends; all sources detected the 2017 seasonal peak for each indicator within a 3-week period. 

The surveillance systems that collect ILI data (Healthdirect and Flutracking) detect more baseline levels of disease activity than those that capture laboratory-confirmed influenza data (the influenza complications alert network and the Australian sentinel practices research network). This is because ILI surveillance detects more activity by capturing symptomatic influenza cases that might not be confirmed and can inadvertently include other respiratory infections. This is reflected in transmissibility and impact parameter figures where the baseline activity is visibly higher among ILI data sources; and in seriousness parameters where the laboratory-confirmed influenza data are less stable early in the season due to low numbers.

Despite the diversity of surveillance systems used, when we applied thresholds to the 2017 season, we found good agreement between data sources in measuring indicator activity levels. In 2017, each parameter within the transmissibility and impact indicators measured the same activity level at the seasonal peak. For disease seriousness, all parameters measured the same activity level at the end of the season, except the age-separated Healthdirect data, which showed circulating influenza had a greater impact on those aged ≥ 65 years. There were some variations, however, in the start and end dates of the 2017 season according to each parameter. Inconsistencies in detecting the epidemic period could be resolved by defining the seasonal period using the first parameter with laboratory-confirmed influenza data to cross its epidemic threshold. For this study, indicator activity was determined simply by a parameter crossing a threshold. However, future consideration should be given to the value placed on the length of time a parameter exceeds a threshold.

The 2017 Australian influenza season was described by the national influenza surveillance committee as having high influenza activity, very substantial absenteeism and health-system burden and low seriousness in all age groups except the elderly (for whom disease was more serious).^20^ This description corresponded with our assessment of activity levels against severity thresholds, which found high transmissibility, extraordinary impact and moderate seriousness in all parameters, except for high seriousness among people ≥ 65 years. While expert opinion is valuable, evidence-based quantification of influenza severity and the epidemic period provides defined, repeatable measurements. Such measurements allow response activities to be planned and instigated, and meaningful comparisons between seasons to be made.

The pandemic influenza severity assessment and moving epidemic method provided useful, flexible methods of selecting and analysing multiple data sources and producing standardized thresholds to assess circulating influenza severity. The recommended threshold for extraordinary disease activity in the pandemic influenza severity assessment method is the upper limit of the one-sided 97.5% CI of the geometric mean.^3^^,^^14^ When we applied this to Australian data, the extraordinary threshold was close to the 2012–2016 seasonal peaks for some parameters. Consequently, we raised the threshold to the upper limit of the 99% CI, which produced more practical thresholds for assessing activity extremes. The method for detecting the optimal timing of the epidemic period within the *memmodel* function was also flexible in providing alternative options for calculation to suit the data. The epidemic curve for Australian influenza data was less steep than the European data that were used to develop the moving epidemic method. This may explain why we needed to use alternative options within the moving epidemic method functions.^6^ The pandemic influenza severity assessment and moving epidemic method are easily tailored to suit the data available in-country, which would enable these methods to be applied in different international surveillance contexts.

The WHO method for measuring disease seriousness was also simple to apply and describes an important facet of influenza severity. As described in the pandemic influenza severity assessment, this indicator is unstable at the beginning of the season and should only be used to assess activity from the seasonal peak onwards.^3^ The seriousness indicator is the most important to examine by age group, as this indicator is relatively stable seasonally, except for differences by age, depending on circulating influenza virus subtypes.^3^ The seriousness indicator is very specific and requires the most limited data. As such, it is recommended that a cumulative rate is used to boost the numbers. This approach was effective in enabling analysis of Healthdirect data by age groups, but ineffective in generating sufficient numbers to enable us to measure seriousness using the influenza complications alert network data. In 2017, when Healthdirect data were assessed as a whole, disease activity was measured as reaching a moderate level of seriousness. Assessment by age group in the same season, however, demonstrated that the older population experienced high seriousness. As such, an overall assessment of seriousness without age separation may mask the effect of a season on a particular group. The low numbers of patients in the surveillance of influenza seriousness highlights the importance of having two data sources to validate severity measurements. Broader data capture is needed in Australia to reliably assess severity by age, particularly for seriousness.

Well-established national surveillance made it simple to implement the pandemic influenza severity assessment in Australia. However, the tool requires consistent, reliable and diverse influenza data from long-term surveillance systems. Whether it is feasible to collect such data in low- and middle-income countries has to be considered. Implementation of the tool is an opportunity to build and guide influenza surveillance capacity. Sentinel surveillance of influenza is the most efficient method of collecting high-quality, low-resource, timely data and is a basic recommendation for WHO Member States.^21^ Sentinel surveillance of ILI and severe acute respiratory illness in primary and secondary health-care facilities (including a population denominator, intensive care unit admissions and available hospital beds) would provide the necessary information to measure all three severity indicators. While health care may be accessed differently in low-resource settings, these measures should provide an indication of influenza severity relative to a country’s recent trends. As such, sentinel influenza surveillance should be prioritized when building capacity in low-and middle-income countries to enable high-value data collection and measurement of indicators.

In addition to the general challenges of influenza surveillance, this study had several limitations. The data sources we used inherently capture different geographic and demographic groups in the population.^4^^,^^16^ A key consideration is the under-representation of residents from the state of Victoria in transmissibility data sources.

We did not measure transmissibility and impact indicators by age group, nor did we investigate the presence of chronic conditions as a subgroup for analysis. Analysis of the seriousness indicator by age groups demonstrated the importance of separation into age groups; analysis by age for all indicators would be desirable. In applying the pandemic influenza severity assessment, a balance must be struck between a country’s need for assessment by age, location and comorbidities and the importance of producing a single, generalized activity level to contribute to global indicator reporting. Although we prioritized creating a national assessment, subgroup analysis should be explored in the future.

Influenza circulates differently in tropical and temperate areas, tending to have multiple epidemic waves and differently timed peaks in tropical regions.^22^^,^^23^

Approximately 40% of Australia’s landmass lies in a tropical climate, however most of the population reside in temperate areas.^24^^,^^25^ National influenza data patterns align with predominant population distribution, and represent a temperate pattern with one defined epidemic wave. As a result, we applied the moving epidemic method’s temperate climate model. The presence of diverse Australian climatic regions, however, should be considered when actions are being recommended based on pandemic influenza severity assessment assessments. Just as separation into age groups is informative as to the distribution of influenza severity, the division of indicators into climatic regions would be useful in countries with diverse weather systems. Availability of data to describe climatic differences should be explored in Australia and internationally.

Our implementation of the pandemic influenza severity assessment demonstrates how the tool can be applied in practice and its usefulness in the Australian context. The agreement across our diverse data sources and the alignment of expert opinion with our threshold measurements shows the tool’s utility in selecting relevant parameters and setting meaningful, consistent thresholds. Routine use and familiarization with the tool will enable measurements to be refined. There is substantial public health benefit in understanding the timing and severity of the influenza season. This can inform responses to seasonal and pandemic influenza, ensuring that funding and prevention and control efforts are directed appropriately. In a season with high transmission levels, symptomatic people can be encouraged to avoid public places. Early detection of a high-impact influenza season could pre-empt extra hospital resourcing. Recognizing a season with high disease seriousness could facilitate targeted prevention activities for those identified as most vulnerable.

On an international scale, having a global view of transmissibility, impact and seriousness patterns can enable early response to influenza pandemics. We hope that this study will encourage other countries to implement the pandemic influenza severity assessment, thereby increasing the strength of international assessment efforts.
